# Revisiting the Identification and cDNA Cloning of T Cell-Replacing Factor/Interleukin-5

**DOI:** 10.3389/fimmu.2014.00639

**Published:** 2014-12-23

**Authors:** Kiyoshi Takatsu

**Affiliations:** ^1^Toyama Prefectural Institute for Pharmaceutical Research, Imizu City, Toyama, Japan; ^2^Department of Immunobiology and Pharmacological Genetics, Graduate School of Medicine and Pharmaceutical Sciences, Toyama City, Toyama, Japan

**Keywords:** B cell differentiation factor, eosinophil differentiation factor, B cell growth factor, TRF, IL-5

## Abstract

This is a perspective based on the paper “*Cloning of complementary DNA encoding T cell-replacing factor and identity with B cell growth factor II*,” by Kinashi et al. (1). We have been interested in understanding the molecular basis of T–B cell cooperation for antibody formation. Although many investigators had described a number of different soluble factors that appeared to have biological relevance to T–B cell interactions, molecular basis of such active substances remained unknown for a long period of time. In this perspective, I will briefly summarize the history of the initial discovery of T cell-replacing factor/B cell growth factor II that appeared to be involved in B cell growth and differentiation, and outline the discovery and characterization of interleukin-5. Studies of interleukin-5 have provided strong evidence that a single cytokine exerts a variety of activities on diverse target cells.

The reciprocal relationship between antibody production and cell-mediated immunity had been well established by 1970. Antigen doses, forms, and routs of administration for stimulating optimum antibody production to T-dependent antigens were found to be usually accompanied by little or no delayed-type hypersensitivity (DTH), and vice versa, despite the fact that both B cell help and DTH were properties of T cells (2). Thus, one of the key issues was how antigen-stimulated T cells regulate both humoral and cell-mediated immune responses. The other issue was how T cells helped B cell differentiation into antibody-secreting cells.

The articles of Rajewsky et al. (3) and Mitchison (4) described the involvement of the cellular cooperation of carrier-specific T cells and hapten-specific B cells for anti-hapten antibody response to hapten-carrier conjugates. Besides the carrier specificity of helper T cells, these cells also strictly recognized MHC class II molecules on antigen-presenting cells. However, in some cases, the interactions between helper T and B cells were not restricted by MHC class II. This was explained by the possibility that both T and B cell populations were functionally heterogeneous. The mechanism underlying B cell triggering in the latter case was interpreted to be mediated by a T cell helper effect via the release of a soluble factor.

Evidence for the existence of such soluble factors came initially from the demonstration in the pioneering studies of Dutton et al. (5), and Schimpl and Wecker (6), both of whom observed that the antibody response of T cell-depleted mouse B cells to the antigens of sheep red blood cells (SRBC) required T cell-derived and macrophage-derived soluble factors detectable in the supernatants obtained from short-term cultures of histoincompatible mouse spleen cells or Concanavalin A stimulation of mouse spleen cells. Although various investigators had described a number of different factors that appeared to have biological relevance to T–B cell interactions, which T cell population secreted such active substances remained unknown. In other words, it had not been established whether those helper T cells that released biologically active mediators belonged to the same T cell population that collaborated with B cells in direct cell-to-cell contact (cognate interactions), or whether these two activities were due to two distinct T cell subsets.

In this review, I will briefly summarize the history of the initial discovery of an activity that appeared to be involved in B cell growth and differentiation, and outline the developments that led to the discovery and characterization of the molecule that was designated interleukin-5 (IL-5).

We had been interested in the adjuvant effects of *Mycobacterium tuberculosis* (Tbc) on both humoral and cell-mediated immune responses. In the early 1970s, we focused on two questions regarding T and B cell cooperation for antibody production: whether a helper T cell subset for B cell activation was also responsible for DTH as effector T cells, and whether T cell-derived factors were involved in B cell activation in the absence of cognate T cell help for antibody production. At that time, the existence of T cell-derived factors that act on B cells in an antigen-non-specific manner was not appreciated.

Since Tbc was well known as an important ingredient of Freund’s complete adjuvant, widely used to enhance antibody production and also to augment DTH responses to a co-immunized particular antigen, we examined tuberculin (PPD)-reactive T cell activity in spleen cells from heat-killed Tbc-primed mice. Helper T cell activity of Tbc-primed cells was assessed by an adoptive cell transfer system in mice by the ability to induce anti-2,4-dinitrophenyl (DNP) IgG antibody-secreting cells from DNP/keyhole limpet hemocyanin (DNP-KLH)-primed mouse B cells. As expected, anti-DNP IgG antibody production was induced by stimulating DNP-primed B cells with DNP-PPD in the presence of Tbc-primed cells (7). However, significant anti-DNP IgG antibody production was also enhanced by stimulating DNP-primed B cells with a DNP-coupled heterologous carrier plus PPD, but only when Tbc-primed cells were present. Furthermore, injection of conditioned medium of PPD-stimulated Tbc-primed cells into recipient mice with DNP-primed B cells augmented the anti-DNP IgG response compared with control supernatants. We tentatively called the active factors produced by T cells as factors that enhanced anti-hapten antibody production.

These findings implied that Tbc immunization of mice could prime two types of helper T cells regarding B cell help; one triggered B cells through direct cell-to-cell contact via the DNP-PPD (cognate interaction) and the other activated their indirect interaction via lymphokine secretion (factor-mediated interaction). We evaluated these possibilities by an *in vitro* culture system, the results of which revealed that Tbc-primed T cells for cognate interaction (Thc) developed as early as 7 days after Tbc-priming and collaborated with T cell-depleted DNP-primed B cells upon stimulation with DNP-PPD. However, Tbc-primed T cells for factor-mediated interaction (Thf) only developed after 4 weeks of priming and could augment the B cell response only in the presence of PPD (8). We further confirmed the existence of two distinct types of helper T cell subpopulations, representative of Thc and Thf, in Tbc-primed cells by establishing two distinctly functioning, long-term-cultured PPD-reactive helper T cell clones. Supernatants of PPD-stimulated Tbc-primed cells enhanced anti-DNP IgG production by DNP-primed B cells in an MHC-non-restricted manner (9). We referred to the enhancing factor(s) as T cell-replacing factor (TRF) because the assay systems for and biological properties of the enhancing factor(s) thus described were similar to those reported by Schimpl and Wecker in 1972 (6). We also found a strain of mice, DBA/2Ha, whose DNP-primed B cells had an X-linked B cell defect, reflected in part by the low responsiveness to TRF, and were unable to be activated through factor-mediated interaction, while they were good responders with Tbc-primed T cells for cognate interaction through DNP-PPD, suggesting the existence of two different subsets in activated B cells (9).

To obtain further insight on the molecular basis of TRF, we took two different approaches. First, we established a monoclonal T cell hybrid clone B151K12 by means of the fusion between Tbc-primed BALB/c T cells and BW 5147 thymoma (10). B151K12, which we referred to as simply B151, produced TRF-active molecules continuously, without stimulation. The cell-free supernatant of B151 could induce an anti-DNP IgG-PFC response in T cell-depleted DNP-primed B cells from various strains of mice. It also restored the primary anti-SRBC IgM PFC response in *nu/nu* spleen cells. As B151 did not produce detectable levels of other cytokines affecting B cell responses, we hypothesized that this B151-derived TRF (B151-TRF) was a novel cytokine, distinct from other cytokines. B151-TRF was able to augment B cell responses in a totally antigen-non-specific manner and in MHC non-restricted manner.

Secondly, we selected a particular clone of *in vivo* growing murine chronic B leukemia (BCL1) cells that preferentially responded to B151-TRF and LPS, and differentiate into polyclonal IgM-secreting cells. BCL_1_ cells appeared to be good target cells for B151-TRF and thus we adopted them for further analysis of TRF-active molecules.

Once B cells are activated, there is an early phase of proliferation in which the responding B cell population expands many times, and a later phase of differentiation induced by TRF in which the already proliferating B cells move on to become Ig-secreting cells. In the early 1980s, several interesting observations were reported indicating that different phases of the B cell response to antigenic stimulation are regulated by functionally and biochemically distinct factors. Howard et al. demonstrated a B cell growth factor (BCGF) in supernatants of PMA-stimulated thymoma cells (designated EL-4) that co-stimulated purified B cells with anti-IgM antibodies in short-term cultures, and induced polyclonal B cell proliferation but not antibody-forming cell generation (11). The BCGF was not mitogenic for resting B cells and interacted with anti-IgM-activated B cells in a non-H-2-restricted manner. The factor was distinct from T cell growth factor (IL-2) and synergized with antigen, IL-2, and IL-2-free, BCGF-free T cell supernatants that contained TRF to generate antibody-forming cells in cultures of highly purified B cells (11). This was the first description of a lymphokine activity (distinct from IL-2) that had the ability to support B cell proliferation, and was tentatively called BCGF (later referred to as BSF-1 and after molecular characterization, IL-4), and the activity eluted at an apparent m.w. of 18 kDa on gel filtration. BCGF(IL-4) was able to synergize with two additional TRF activities to trigger B cells to Ig-secreting cells; one was a TRF-containing cell-free supernatant from B151, and the other was a TRF activity derived from PMA-stimulated EL-4 cells. However, it was not clear whether these two TRF activities were identical or distinct.

Concomitantly, Swain and Dutton reported a second BCGF with an apparent m.w. of 50 kDa on gel filtration in a cell-free supernatant from an IL-2-dependent T cell line (designated Dennert) that induced the proliferation of dextran sulfate-stimulated B cells or BCL_1_ cells, whereas it did not induce anti-IgM-stimulated B cells to proliferate (12). The 18 kDa BCGF described by Howard et al. was designated as BCGFI and the 50 kDa BCGF as BCGFII. Soon after that, Swain found that their BCGFII preparation was able to induce Ig-secretion from activated B cells and that this differentiation activity co-purified with the proliferative activity in a variety of chromatographic separations. We demonstrated that B151-TRF could induce not only differentiation but also the proliferation of B cell populations that were co-stimulated with anti-IgM antibody plus BCGFI. Swain and Dutton independently demonstrated that B151-TRF promoted the proliferation of DXS-stimulated B cells or BCL_l_ cells, suggesting that B151-TRF exerted BCGFII activity on pre-activated B cells. An important question became whether B151 cells produced both TRF and BCGFII or whether a B151-TRF molecule also exerted BCGFII activity.

When we started to purify TRF-active molecules to homogeneity, we were in the front line among TRF-hunting groups. However, concepts of BCGFI and BCGFII for B cell growth and differentiation had by this time become accepted by most workers in the field. So, we had to clarify whether B151-TRF was identical to either BCGFI or BCGFII. We examined the BCGFI activity of our purified B151-TRF using anti-IgM-stimulated B cells and found that B151-TRF did not show detectable levels of BCGFI activity. However, we independently confirmed the activity of B151-TRF on BCL_1_ cells, thereby defining that it had BCGFII activity (13).

Our strategy for identifying TRF(BCGFII) was to first purify TRF-active molecules to homogeneity from the cell-free supernatant of B151, including the identification of its partial NH2-terminal amino-acid sequence. Overall, B151-TRF was purified approximately 1,400,000-fold, and the activity could be attributable to an extremely hydrophobic glycoprotein with a molecular mass of 40–60 kDa and a smaller mass (25–30 kDa) under reducing conditions on gel filtration. Highly purified B151-TRF also showed BCGFII activity, whereas it did not exert detectable IL-1, IL-2, IL-3, (BCGFI)IL-4, or IFN-γ activities. During each purification step, the BCGFII activity could not be separated from the TRF activity and always resided in the same fraction in which the TRF activity was detected (13).

Although we attempted to determine the partial NH2-terminal amino-acid sequence of purified B151-TRF several times, we were unsuccessful. So, we immunized rats with purified B151-TRF and their spleen cells were fused with mouse myeloma cells to generate monoclonal antibodies (mAb) reactive with B151-TRF. We obtained two different anti-mouse TRF mAb, designated as NC17 and TB13, which neutralized the TRF and BCGFII activities of B151-TRF, and blocked B151-TRF-induced IgM secretion and the proliferation of BCL_1_, whereas they did not inhibit activities of IL-1, IL-2, IL-3, (BCGFI)IL-4, and IFN-γ (14). Immobilized mAb TB13 as well as NC17 proved feasible for reproducibly purifying TRF-active molecules from the conditioned medium of B151 and PPD-stimulated Tbc-primed cells with molecular weights of approximately 50 kDa under non-reducing conditions (15).

To obtain concrete evidence that the molecular properties of mouse TRF and BCGFII were in fact identical, we isolated a cDNA encoding TRF from a 2.19 T cell line by using a pSP6K cDNA library, in collaboration with Honjo’s group. Since structural characterization was not available at that time, we felt that the best strategy was to use an expression vector system that required only a limited amount of mRNA. We decided to apply and construct a new expression vector system, pSP6K containing the SP6 promoter (16). With the aid of a specific RNA polymerase, the pSP6K vector allowed the synthesis of up to a few micrograms of poly A^+^ RNA by *in vitro* transcription. This mRNA was directly injected into *Xenopus* oocytes and the oocyte culture supernatants were analyzed for their biological activities. In this way, secretory proteins could be synthesized in a system that was mycoplasma-free, free of serum proteins, and highly concentrated.

As cultured supernatants of the 2.19 T cell line exerted TRF activity like B151-TRF, poly A^+^ RNA of 2.19 T cells was fractionated and enriched by sucrose gradient fractionation and aliquots of RNA were microinjected into *Xenopus* oocytes and the oocytes’ culture supernatants were analyzed for TRF and BCGFII activities (1). cDNA libraries from polyA^+^ RNA and from the mRNA enriched by the sucrose gradient fractionation were constructed using the pSP6K vector system. Pools that scored positive in the biological assays were further divided into smaller pools that were analyzed in the same manner until single cDNA clones capable of directing the synthesis of biologically active TRF preparations were obtained. Finally, pSP6K-mTRF was shown to encode TRF activity (1). The isolated cDNA clones were then subjected to nucleotide sequencing analysis.

We obtained a cDNA clone, pSP6-mTRF23, which after hybrid selection of mRNA, a translation product was obtained that showed both TRF and BCGFII activities (1). Both TB13 and NC17 mAbs neutralized TRF and BCGFII activities of products secreted by *Xenopus oocytes* that had been injected with mouse TRF mRNA transcribed from plasmid pSP6K-mTRF23 (17). The primary sequence of TRF deduced from the cDNA revealed a novel interleukin-type molecule consisting of 133 amino acids. The putative secreted core polypeptide has a relative m.w. of 12.3 kDa that is close to that of B151-TRF (18 kDa) after glycosylation. Recombinant TRF was also found to be a dimer; reduced and alkylated recombinant TRF lost both TRF and BCGFII activities. These properties of recombinant TRF agreed with the criteria derived from B151 cells. We determined the partial NH2-terminal amino-acid sequence of 13-residues of immunoaffinity-purified recombinant TRF and B151-TRF, and found that they were nearly identical (17). A single amino-acid sequence of each sample obtained beginning from methionine was identical to that predicted from our cDNA, supporting the notion that secreted TRF polypeptide consists of 113 amino acids.

Although TRF was initially believed to be principally active on B cells, recombinant TRF was shown to increase the expression of IL-2 receptor on antigen-stimulated thymocytes, inducing them into cytotoxic T lymphocytes (CTL) in the presence of IL-2. TRF also induced the terminal differentiation of hematopoietic progenitor cells into eosinophils, besides the activities on activated B cells (17). As TRF was the fifth interleukin activity for which the primary structure had been determined, we proposed that TRF and BCGFII be called interleukin-5 (IL-5) (1). The molecular cloning of cDNA encoding both murine and human IL-5 convincingly demonstrated that a single molecule was responsible for both TRF and BCGFII activities. However, in humans, the major target cells for IL-5 are eosinophil myeloid progenitors and mature eosinophils. Human peripheral blood B cells do not respond to human IL-5 by differentiation to Ig-secreting cells. Consequently, the human B cell growth and differentiation interleukins for peripheral blood B cells appeared to be IL-2, IL-4, and IL-6 (18, 19). Subsequently, as time progressed, several new cytokine molecules would be described that influence B cells in addition to these first three discovered.

Studies on IL-5 have provided strong evidence that a single lymphokine exerts a variety of activities on diverse target cells. Together with similar properties of IL-4, these results helped many immunologists escape earlier beliefs that one lymphokine is required for each activity and that growth factor activity and differentiation factor activity are necessarily different molecular entities.

I focused on the historical perspective of the discovery of T cell derived IL-5 that promoted B cell growth or differentiation. Around the same time, largely between 1985 and 1987, there were parallel efforts by a number of groups working on eosinophil differentiation [e.g., Ref. (20)] and T cell factors that enhanced IgA production [e.g., Ref. (21)] that ultimately also led to purification and identification of IL-5.

## Conflict of Interest Statement

The author declares that the research was conducted in the absence of any commercial or financial relationships that could be construed as a potential conflict of interest.
